# Psychological Well-Being in Teachers During and Post-Covid-19: Positive Psychology Interventions

**DOI:** 10.3389/fpsyg.2021.769363

**Published:** 2021-12-16

**Authors:** Diego García-Álvarez, María José Soler, Lourdes Achard-Braga

**Affiliations:** ^1^Jóvenes Fuertes Uruguay, Montevideo, Uruguay; ^2^Departamento de Ciencias del Comportamiento, Universidad Metropolitana, Caracas, Venezuela; ^3^Centro de Estudios de Psicología, Universidad de Montevideo, Montevideo, Uruguay; ^4^Consejo de Formación en Educación, Administración Nacional de Educación Pública, Montevideo, Uruguay

**Keywords:** psychological well-being, teachers, positive psychology, positive psychology intervention, subjective well-being

## Introduction

One of the major consequences of Covid-19 in educational settings has been the transition from face-to-face instruction to emergency remote teaching in order to maintain teaching and learning quality standards (United Nations Educational Scientific Cultural Organization, 2020; Garcia et al., 2021). Studies have shown a marked increase in stress and burnout in school teachers (Pellerone, 2021) and technostress among university teachers during the pandemic as a result of different reasons both subjective and objective (Penado-Abilleira et al., 2021), moderate to low levels of stress, anxiety and depression in school and university teachers (Ozamiz-Etxebarria et al., 2021a). Fear of contagion and risk perception in the school context, and teacher with higher levels of anxiety considered that the possibility of reopening schools was not a priority (Weinert et al., 2021). A systematic review carried out on teacher health in times of Covid-19 (Holguín, 2021) has found that physical, mental and social health have been impacted. It is relevant to mention that only two studies were found related to this. One was about interventions for the prevention of physical symptoms (Kayabinar et al., 2020) and the other about emotional competencies (Roman, 2020).

Non-university teachers have reported an increase in psychosocial risks in working environments regarding limited resources, difficulties in organizational justice, interpersonal problems, role confusion and work overload, uncertainty management, psychosomatic disorders, and burnout as well as the responsibility of being the primary learning facilitator of children and teenagers (Prado-Gascó et al., 2020). One of the concerns expressed by teachers has involved assessments, including assessment tools and strategies, monitoring student learning and ethical issues related to students' behaviors (Jelińska and Paradowski, 2021).

In addition to other factors determined by education administrations, vaccination plans in some parts of the world have contributed to the gradual return to face to face instruction or at least a hybrid model of education. Nevertheless, teachers who have faced the prospect of going back to onsite teaching have experienced anxiety about contagion risk as well as falling behind or having difficulty keeping up with the planned teaching schedule and overall student progress (Wakui et al., 2021). Teachers who have returned to face-to face lessons have reported high levels of anxiety, stress, and even depression, all of which were likely exacerbated by the emotional experience they have gone through during the lockdown period, the uncertainty about contagion in schools and managing their workload from home (Ozamiz-Etxebarria et al., 2021b).

Teaching staff are not coming back to what was considered a “normal” school environment before the pandemic breakout (Darling-Hammond and Hyler, 2020; Ellis et al., 2020; Brunzell et al., 2021; Pressley, 2021a). Educational institutions are reopening after high levels of Covid-19 and with a current marked increase in variants such as Delta. In this context, it is worth considering that we may be coming back to school experiencing collective psychosocial trauma (Bergren, 2021; Gonçalvez-Boggio, 2021). A scenario that promotes teacher burnout (Pressley, 2021b). With the current educational situation, we must reflect upon how to promote teachers' psychological well-being both in the present-day and post-Covid-19.

In order to identify the research on teachers' psychological well-being in the context of the COVID-19 pandemic, a systematic review was carried out following the PRISMA method (Moher et al., 2009). Following each of the phases, a comprehensive literature search was conducted in three databases: SCOPUS, Web of Science (all collections) and EBSCO (all collections), using the keywords “wellbeing” OR “well being” OR “well-being” AND “teacher” OR “teacher” AND “positive psychology” AND “covid-19” OR “coronavirus” OR “2019ncov” OR “sars-cov-2” OR “cov-19” identified anywhere in the registry. The search for primary sources included only English and Spanish language scientific articles. The initial search yielded four results.

After discarding duplicate works, a total of three studies were identified. The analysis of each of them resulted in: (a) a qualitative research about the trauma approach in conjunction with applied positive psychology for elementary school teachers (Brunzell et al., 2021); (b) a study focused on coping strategies in relation to well-being, stress and negative emotions in language teachers at different teaching levels (MacIntyre et al., 2020); and (c) a theoretical proposal focused on methods and teaching strategies based on well-being and positive psychology for teachers but focused on student well-being (Chu, 2020).

Given the few studies found in the context of the COVID-19 pandemic, the intention of this article, which focuses its efforts on the contribution of positive psychology to teachers' well-being, is relevant. For this purpose, a new systematic review was conducted following the steps described above. However, the descriptors associated with COVID-19 were substituted by research oriented to applications or intervention programs (“program” OR “intervention”). In this case, 89 articles were found in the three databases consulted. Twenty-six of these were duplicates.

The titles and abstracts of 63 articles were read. The selection criteria were: (1) research focused on teachers, (2) positive psychology topics, (3) in English or Spanish. The final sample consisted of 12 research studies. We identified 66.7% (*n* = 8) quantitative paradigm studies, 33.3% (*n* = 4) with a qualitative design. Only 16.6% (*n* = 2) of them were published in Spanish. The findings highlight the fact that, although the rise of positive psychology applied to education has been important in recent years, the work developed has a greater emphasis on the context of student well-being. There is little research that considers the teacher from a person-centered approach, despite being an essential actor for the development of positive education (Rahm and Heise, 2019; White, 2021). Perspectives offered by Positive Psychology provide us with some important answers. Please note that references to “teaching staff” in this article refer to teachers working at different formal educational levels including preschool, primary, secondary, and university or higher education.

## Positive Psychology in Teachers' Well-Being

Teachers' well-being is a complex construct, which has been conceptualized as the absence of negative conditions such as teacher's stress, demotivation and even burnout (Huertas and Dávila, 2020; Bastías, 2021). Teachers' well-being has also been studied as it relates to coping strategies and engagement and recovery from work (Pöysä et al., 2021). From the perspective of Positive Psychology, well-being may be analyzed with a holistic approach such as Seligman's PERMA model (Seligman, 2012) as well as in eudaimonic dimensions such as the multidimensional psychological well-being model proposed by Ryff (1989, 2014). It is important to understand that well-being is a complex construct that includes a variety of subjective indicators. Some of which are related to personal growth and self-actualization, which must always be considered in context. Research measuring teachers' psychological well-being has yielded interesting results. These may suggest guidelines for the development of psychosocial intervention strategies.

When focusing on psychosocial interventions, the strategies to be developed may better fit into an approach that favors health promotion, and prevention risk factors along with primary prevention of psychopathology. These may be universal interventions targeting all teaching staff. From a theoretical point of view, these interventions aimed at increasing the protective factors for mental health and well-being of teachers (Branand and Nakamura, 2017) are based on the Job Demands-resources Model (Bakker and Demerouti, 2013), the Theory of Flow and Optimal Experience (Csikszentmihalyi, 1988), the Classification of Character Strengths and Virtues (Peterson and Seligman, 2004), the Social Cognitive Career Theory (Lent et al., 1994), and the Self-determination Theory (Ryan and Deci, 2017).

Furthermore, previous research involving teachers at different educational levels has identified factors affecting teachers in the workplace. These should be considered when addressing risk reduction and burnout prevention (Carlotto and Câmara, 2017; Tabares-Díaz et al., 2020). The above mentioned factors include interpersonal variables such as emotional expression and regulation, motivation, self-efficacy, and teacher engagement (García-Renedo et al., 2006; Perandones-Gonzalez et al., 2014; Lozano-Paz and Reyes-Bossio, 2017).

In addition, context-bound variables related to peer support and collaboration, school work environment, school leadership and management, and the impact of public-school policies (Dávila, 2018) should also be considered. In the field of Positive Psychology, specific interventions aimed at promoting multidimensional psychological well-being have been implemented (e.g., multidimensional well-being workshops for teachers, Leal-Soto et al., 2014). Particular character strengths such as gratitude (e.g., counting blessings, Chan, 2010) can decrease symptoms of depression in teachers and increase their level of satisfaction (e.g., counting blessings vs. misfortunes, Chan, 2011, 2013). Mindfulness interventions may be used to strengthen personal resources, thereby reducing work stress among teachers (Taylor et al., 2015).

In the research laboratory of Jóvenes Fuertes Uruguay, multicomponent interventions aimed at promoting psychological well-being in teachers have been developed. The goals of the Positive Psychology Course Applied to Education (CUPPAE according to its acronym in Spanish) were to increase the psychological well-being of educational staff through the identification of their virtues and character strengths, and to provide training in teaching strategies to apply positive psychology in their classrooms. The study had a pretest–posttest design with teachers of various educational levels.

The intervention consisted of eight modules providing a formative journey through positive psychology, elements of well-being, character strengths, positive education and strategies, mental styles, resilience and optimism, mindfulness in conjunction with self-regulation, emotional management, empathy and compassion. The results indicated a significant increase in psychological well-being in each of its dimensions in the participating teachers before and after the intervention. It was concluded that the intervention presented satisfactory preliminary results (García-Álvarez et al., 2020).

The Jóvenes Fuertes research laboratory has developed an additional multi-component positive psychology intervention aimed at developing multidimensional psychological well-being and gratitude. This intervention uses a pre- and post-measurement methodology with a single group, and comprises eight sessions. In this intervention, teachers start from a Positive Psychology foundation, well-being models, and focus on each of the character strengths including an organized gratitude campaign in the school environment. Pre and post-tests results have indicated a marked increase in teachers' psychological well-being and gratitude. It was concluded that the intervention could be used to promote psychological well-being, gratitude and mental health in teachers (García-Álvarez and Soler, 2021).

The results of both studies suggest two significant findings. First, regarding continuous professional development, the main objective of the intervention must be to train teachers to apply positive psychology both in their own lives and to implement its principles in the classroom. Second, regarding the nature of the psychological intervention, the mental health of the teachers and the educational community as a whole must be promoted. The results of these studies provide recommendations indicating that any positive psychology intervention designed for teachers should integrate all aspects, including teaching practice, educational leadership, and school management. Similarly, these interventions should address specific situations related to teacher distress, as indicated by the prevention model in Keyes (2002) two-factor model (Kern et al., 2014; Brunzell et al., 2021; World Health Organization, 2021).

## Teacher Well-Being During and After COVID: Ideas From Adversity

The Covid 19 pandemic will continue to present challenges to the quality of education at many different levels. The following discussion by the authors suggests possible interventions or guidelines to maintain and promote teachers' well-being during a difficult time. According to Waters (2021), actions taken to promote school well-being should be systematized and based on scientific data to gather empirical evidence that will eventually be reported in academic or professional environments.

The authors promote goals to empower teachers through teaching practices that integrate wellness practices into their curricula. Creating interventions with a context-based approach that will foster skills to increase well-being in the classroom (e.g., Waters, 2021). Social relationships and connections are the building blocks of multidimensional well-being models. Relationships between teachers and students are paramount in the development of well-being and these have a special importance during the Covid-19 pandemic. Studies have revealed the relevance of the interaction between teachers and students in an online teaching environment (Alqurshi, 2020; Bao, 2020; Chanchí-Golondrino et al., 2021; Hamdan et al., 2021; Jelińska and Paradowski, 2021).

Consequently, it is important that interventions aimed at promoting teacher well-being include practical strategies to improve interpersonal interactions. These include emotional intelligence, empathy, assertiveness, compassion, etc., which can influence the socioemotional classroom environment and improve relationships between colleagues and teaching peers. Psychological working resources that enhance reflective teaching practices and improve teaching and learning should be promoted. These would include character strengths such as gratitude, creativity, love of learning, bravery, and others (White, 2021). Fostering teacher autonomy in decision making may be achieved with institutional support and a respectful leadership based on mutual trust and adjusted to different working conditions (Naegeli Costa et al., 2021).

The authors identify the importance of promoting flow experiences, which will lead to lasting engagement to teaching experiences as they create lesson plans and classroom materials, deliver lessons and other teaching duties. These flow experiences may prove to be protective factors against stressors, burnout, and teacher drop out (Millán de Lange et al., 2014). The authors stress the essential inclusion of a trauma informed care focus in well-being interventions for teachers during and post-Covid (Brunzell, 2021) as the pandemic may be experienced as a psychosocial trauma by the teachers (Gonçalvez-Boggio, 2021). This trauma-informed care perspective may be useful for teachers to help themselves and to better understand other members of the educational community that may need support in the re-adaptation process.

One of the proposals is the implementation of Professional Development Teacher Communities (Webb et al., 2009; Vaillant, 2019; García-Álvarez, 2020) focused on well-being as a systematic way to expand the knowledge oriented to the continuous development of teachers, both at a personal and professional level to improve the quality of education. In this regard, the role of educational leadership is key to implementing this initiative. In addition to strategic alliances with other specialists inside and outside the school such as psychologists, counselors, etc., it is important to consider the overlap between sectors either inside or outside the educational system. For example, the areas of labor and health (Karaman et al., 2021).

The benefits of this initiative will be the improvement of pedagogical practices, reconfiguration of professional teaching competencies, increased recognition of teamwork and the organization of work teams. The advice and help of teaching leaders and colleagues will be sought and examples of good educational practices identified. A systematic intervention oriented to medium and long-term outcomes must be framed with quality educational management and with the school as a construction of well-being spaces, a center for promoting mental health (García-Álvarez et al., 2021).

The professional teacher learning community can mobilize the factors that generate organizational learning with a clear management commitment open to continuous learning and a climate that promotes learning. Members of the educational community must be located in an environment that favors training and the exchange of experiences, and must have an infrastructure that allows the educational organization to function optimally in all its aspects. Valid and reliable psychometric scales to assess constructs related to teacher well-being should be included in any proposed study (e.g., Renshaw et al., 2015). Through this initiative, teacher empowerment would be promoted by involving teachers in decisions (Yusoff and Tengku-Ariffin, 2020). In conclusion, the authors call for the design of public policies for teacher training, professional development and retention in the education system to face the challenges ahead. Challenges that the political and educational actors of the system must assume with leadership.

## Author Contributions

DG-Á: formulation of the idea, initial and final writing, and constant revision. MS and LA-B: reviews, contributions, and final writing. All authors have contributed to the work and have approved the final version submitted.

## Funding

The authors declare that this study received funding from Jóvenes Fuertes Uruguay. The funder was not involved in the study design, collection, analysis, interpretation of data, and the writing of this article or the decision to submit it for publication.

## Conflict of Interest

The authors declare that the research was conducted in the absence of any commercial or financial relationships that could be construed as a potential conflict of interest.

## Publisher's Note

All claims expressed in this article are solely those of the authors and do not necessarily represent those of their affiliated organizations, or those of the publisher, the editors and the reviewers. Any product that may be evaluated in this article, or claim that may be made by its manufacturer, is not guaranteed or endorsed by the publisher.
